# Echocardiographic effects of medetomidine, butorphanol, and their combination in donkeys

**DOI:** 10.1186/s12917-026-05678-3

**Published:** 2026-07-04

**Authors:** Marwa Abass, Mohamed Hamed, Helmy K. Elnafarawy, Alshimaa M. Farag

**Affiliations:** 1 Department of Surgery, Anesthesiology, and Radiology, Faculty of Veterinary Medicine, Mansoura University, Mansoura, 35516 Egypt; 2 Department of Surgery, Anesthesiology and Radiology, Faculty of Veterinary Medicine, Aswan University, Aswan, Egypt; 3 Department of Internal Medicine and Infectious Diseases, Faculty of Veterinary Medicine, Mansoura University, Mansoura, 35516 Egypt

**Keywords:** Butorphanol, Cardiac performance, Donkey, Medetomidine, M-mode standing sedation

## Abstract

**Background:**

Standing sedation is frequently required in donkeys for minor surgical and diagnostic procedures, yet information on the cardiac safety of α2-adrenoceptor agonists and opioid combinations in this species is limited. This study evaluated the echocardiographic effects of intravenous medetomidine, butorphanol, and their combination in clinically healthy donkeys.

**Materials and methods:**

Sixty donkeys were randomly assigned to four groups (*n* = 15/group) to receive intravenous saline (control group, CG), medetomidine group (10 µg/kg, MG), butorphanol group (50 µg/kg, BG), or medetomidine–butorphanol group (10 µg/kg + 50 µg/kg, MBG). Butorphanol was administered 5 min after medetomidine in the MBG. M-mode echocardiography was performed from a right parasternal short-axis view at baseline and at 5, 15, 30, 45, 60, 90, and 120 min after treatment. Left ventricular internal diameter (LVID), interventricular septal thickness (IVST), and left ventricular posterior wall thickness (LVPW) were measured at end-diastole (d) and end-systole (s). Left ventricular end-diastolic volume (LVEDV), left ventricular end-systolic volume (LVESV), stroke volume (SV), ejection fraction (EF%), and fractional shortening (FS%) were calculated. Data were analyzed using a repeated-measures general linear model.

**Results:**

Medetomidine alone was associated with significant reductions in EF% and FS%, together with significant changes in LVIDs and LVIDd. SV was also significantly lower in the medetomidine group than in the control group during the main post-treatment period; however, SV and the calculated LV volumes were interpreted cautiously because they are load-dependent variables derived from linear M-mode measurements. These changes were accompanied by an increase in LVIDs and a reduction in IVSs, indicating transient depression of conventional left ventricular systolic indices and altered loading conditions. Butorphanol alone produced only minor, parameter-specific changes in LVID, IVST, and most echocardiographic indices, with no consistent clinically relevant deterioration compared with baseline or the CG. In contrast, the MBG showed marked changes in ventricular dimensions and calculated indices from 15 to 60 min; however, these findings were interpreted cautiously because the calculated systolic indices are load-dependent.

**Conclusions:**

Intravenous medetomidine, butorphanol, and their combination exert distinct and protocol-dependent effects on left ventricular dimensions and systolic function in donkeys. Medetomidine alone substantially and transiently depresses systolic performance, whereas butorphanol alone is comparatively cardiovascular-sparing. The medetomidine–butorphanol combination may be considered for standing sedation in clinically healthy donkeys; however, its echocardiographic effects should be interpreted cautiously as load-dependent changes, and cardiovascular monitoring remains advisable.

## Introduction

Working donkeys, particularly those employed by Egyptian farmers, are exposed to harsh environmental and management conditions, including long working hours, heavy loads, malnutrition, inappropriate harnesses, and limited veterinary care, which markedly compromise their health and welfare [1–3]. These welfare and management challenges may increase the need for safe sedation protocols in working donkeys, particularly because underlying disease may be clinically under-recognized in field conditions. Cardiovascular disease in donkeys is most commonly related to acquired valvular disease, particularly aortic regurgitation or insufficiency, and less frequently to congenital defects [4, 5]. Although post-mortem observations suggest that cardiovascular disease may be under-recognized clinically in donkeys [5], available field data indicate a low prevalence of audible cardiac murmurs [4]. In a cross-sectional survey, murmurs were detected in approximately 2% of donkeys, with findings largely consistent with acquired valvular disease (notably aortic regurgitation) and occasional congenital defects [4].

In clinical practice, standing sedation is commonly employed in donkeys to facilitate procedures such as dentistry, castration, wound management, and other minor surgical or diagnostic interventions, particularly in working equids managed in low-resource field conditions [6, 7]. Standing sedation is frequently achieved using α2-adrenoceptor agonists, alone or combined with opioids to enhance analgesia and deepen sedation. Donkeys differ from horses in pharmacokinetics and clinical responses to sedative and analgesic agents [8, 9]. Importantly, a growing body of donkey-specific literature has evaluated the use of α2-adrenoceptor agonists and opioids in this species and should therefore be considered independently from horse-based data.

α2-adrenoceptor agonists have been shown to produce drug- and dose-dependent sedative and antinociceptive effects in donkeys, with measurable differences among agents. Comparative studies have demonstrated significant variation in the mechanical hypoalgesic effects of different α2-agonists in this species [10]. In addition, dexmedetomidine has been shown to induce dose-dependent sedation and mechanical antinociception in donkeys [11].

In addition, the echocardiographic effects of α2-adrenoceptor agonists such as xylazine and dexmedetomidine following epidural administration have also been described in donkeys [12], further supporting the availability of species-specific cardiovascular data in this species. Opioids such as butorphanol have also been investigated in donkeys, both alone and in combination with α2-agonists. In donkeys, butorphanol has been investigated both alone and in combination with α2-adrenoceptor agonists. It contributes to sedation and mechanical hypoalgesia in xylazine-premedicated donkeys [13], while pharmacokinetic studies confirm species-specific drug disposition in this species [14]. Collectively, these findings indicate that medetomidine, dexmedetomidine, and butorphanol-based protocols in donkeys cannot be directly inferred from horse data and warrant dedicated investigation in this species.

Among the α2-adrenoceptor agonists used in equids, medetomidine is a potent and selective α2-adrenoceptor agonist with greater affinity for α2:α1- adrenoceptor receptors than xylazine [15]. It produces dose-dependent sedation, analgesia, and muscle relaxation and allows smooth induction and recovery from anesthesia. Across species, medetomidine has been associated with marked cardiovascular effects, including bradycardia, hypertension, hypotension, reduced myocardial perfusion, and arrhythmias.

Butorphanol is widely used in equine clinical practice because it provides analgesia and enhances the sedative effects of α2-adrenoceptor agonists when used in combination protocols. Therefore, donkey-specific evidence supports the use of butorphanol-based protocols rather than relying solely on horse-derived data.

In donkeys, transthoracic echocardiography (two-dimensional, guided M-mode and Doppler) provides a repeatable, non-invasive assessment of cardiac chamber dimensions, systolic function, and valvular regurgitation. Standardized imaging and measurement approaches have been described in horses [16, 17].

Echocardiography is also widely used in preclinical drug toxicology and safety pharmacology to detect drug-related changes in cardiac morphology and performance [18]. Importantly, sedative drugs can themselves modify echocardiographic variables; [19] demonstrated that detomidine—and, to a lesser extent, romifidine—significantly altered left ventricular internal diameter at end-diastole (LVIDd) and left ventricular internal diameter at end-systole (LVIDs), fractional shortening (FS), and the occurrence of valvular regurgitation in clinically normal horses, underscoring that sedation can influence echocardiographic interpretation. Comparable controlled data describing these sedation-related echocardiographic changes are currently limited in donkeys.

Although donkey-specific echocardiographic data are available in the form of published reference values/intervals for clinically healthy donkeys [20, 21], these reports primarily define baseline measurements rather than drug-induced, time-dependent changes. In contrast, most evidence regarding the cardiovascular effects of α2-agonists and opioid combinations is extrapolated from horses or focuses on clinical/hemodynamic variables, with limited serial echocardiographic assessment in donkeys. Therefore, there remains an evident lack of donkey-specific data on the time course of echocardiographic chamber dimensions and systolic function indices following intravenous medetomidine and/or butorphanol administration in clinically healthy standing donkeys.

Therefore, we hypothesized that medetomidine would alter conventional systolic indices and that butorphanol co-administration would modify these effects in a load-dependent manner. Consequently, the objective of the present study was to evaluate and compare the echocardiographic effects of intravenous administration of medetomidine, butorphanol, and their combination in clinically healthy standing donkeys.

## Materials and methods

All experiments were performed in accordance with relevant guidelines and regulations, including the ARRIVE guidelines [22]. Ethical approval details are provided in the “Ethics approval and consent to participate” section.

### Animals and sample size

Sixty adult castrated male donkeys, clinically healthy based on physical and cardiovascular examination, aged 6 ± 1 years and weighing 180 ± 50 kg, were used in this study. Values are expressed as mean ± standard deviation (SD).

The donkeys were equally allocated into four independent treatment groups (*n* = 15 per group): control group (CG), medetomidine group (MG), butorphanol group (BG), and medetomidine–butorphanol group (MBG). A sensitivity power analysis was performed using G*Power for a one-way ANOVA design with four groups. The analysis indicated that a total sample size of 60 donkeys would provide 80% power at an alpha level of 0.05 to detect a large effect size (Cohen’s f = 0.44). Repeated measurements were obtained over time within each group.

Before the trials, each animal underwent a complete clinical and cardiovascular examination. This included assessment of general physical condition, rectal temperature, mucous membrane color, capillary refill time, heart rate, cardiac rhythm, intensity and quality of heart sounds, and careful thoracic auscultation to detect abnormal heart sounds or cardiac murmurs. No animals were excluded from the study, and all enrolled animals completed the serial echocardiographic assessment up to 120 min and were subsequently monitored for 12 h for adverse events, with no protocol deviations or complications recorded.

All donkeys were housed individually under standard management conditions at the Veterinary Teaching Hospital, Faculty of Veterinary Medicine, Mansoura University, Mansoura, Egypt. They were fed a maintenance-balanced mixed diet with free access to water. Fourteen days prior to the study, all donkeys were dewormed with Equiveen Paste (ivermectin paste, 0.2 mg/kg, per os; Adwia Company, Egypt).

### Study design

A parallel-group design was selected after considering both the principle of Reduction and the potential for carry-over or period effects. Although a crossover design can reduce the number of animals used and may be appropriate when reliable pharmacokinetic and pharmacodynamic washout data are available, the primary outcomes in the present study included cardiovascular function and echocardiographic indices, which may be influenced by residual drug effects, repeated sedation, repeated restraint, catheterization, and time-related physiological adaptation. Therefore, each donkey received only one treatment to avoid within-animal carry-over effects and to preserve the independence of treatment comparisons. To improve statistical efficiency while limiting animal use, repeated measurements were obtained over time within each animal, and the sample size was supported by power analysis.

### Experimental procedures

This was a prospective, randomized, controlled experimental study. After aseptic placement of a 14-gauge, 48-mm catheter into the left jugular vein, donkeys were assigned to one of four treatment groups by simple randomization using sealed opaque envelopes: control group (CG), medetomidine group (MG), butorphanol group (BG), or medetomidine–butorphanol group (MBG).

Donkeys in the control group received intravenous normal saline (0.9% sodium chloride injection, USP; Hospira, Lake Forest, IL, USA). Donkeys in the MG received medetomidine (10 µg/kg IV [23]; Domitor^®^; Zoetis, Parsippany, USA), while donkeys in the BG received butorphanol (50 µg/kg IV [24]; Nargesic^®^; ACME Srl, Reggio Emilia, Italy). Donkeys in the MBG received medetomidine (10 µg/kg IV), followed by butorphanol (50 µg/kg IV) immediately after completion of the T5 echocardiographic recording; therefore, subsequent time points were used to assess the combined effect of medetomidine and butorphanol.

The final volume of each treatment was adjusted to 10 mL using normal saline. Blinding of the examiner to treatment allocation was not feasible because of the observable sedative effects of the administered drugs. To minimize assessment bias, the echocardiographic protocol, image acquisition procedures, and measurement methods were standardized across all groups.

#### Echocardiographic examination

Echocardiographic measurements were obtained at baseline (T0) and at 5, 15, 30, 45, 60, 90, and 120 min after the initial treatment administration (T5, T15, T30, T45, T60, T90, and T120). Between consecutive echocardiographic examinations, donkeys remained quietly in the examination area until the next scheduled measurement. No additional drugs, procedures, exercise, or interventions were performed between measurements. At baseline, all donkeys were examined without sedation. Subsequent echocardiographic examinations were performed after administration of the assigned treatments, with no additional sedative or tranquilizing drugs administered during the study period. The hair coat was clipped over the right 4th and 5th intercostal spaces, just dorsal to the olecranon and caudal to the triceps muscle. Each donkey was positioned standing alongside the examiner with the right thoracic limb slightly advanced and abducted. Acoustic coupling was achieved with ultrasound gel.

A baseline transthoracic echocardiographic examination was performed for each donkey at T0 to confirm the absence of clinically relevant structural or functional cardiac abnormalities and to verify that key B- and M-mode measurements and derived functional indices were within the published reference intervals for clinically healthy standing donkeys [20, 21]. All echocardiographic image measurements were carried out in accordance with the recommendations for cardiac chamber quantification issued by the American Society of Echocardiography and the European Association of Cardiovascular Imaging [25].

Echocardiographic examinations were performed using a CHISON (iVis 60 EXPERT VET; CHISON Medical Imaging Co., Ltd., Wuxi, China) Doppler ultrasound system with a phased-array transducer (2.0–3.9 MHz). All examinations were digitally recorded and analyzed offline. Dimensions were measured to the nearest 1 mm using the inner edge method. M-mode tracings were obtained from a standard right parasternal short-axis view at the chordal level, with the M-mode cursor positioned perpendicular to the interventricular septum and left ventricular posterior wall. Cardiac indices, including FS%, EF%, and SV, were calculated from left ventricular internal dimensions using the Teichholz method. Measurements were obtained from digitally stored images and averaged over three non-consecutive cardiac cycles to minimize beat-to-beat variability [16].

#### Echocardiographic measurements (M-Mode)

##### Cardiac dimensions

M-mode tracings of the left ventricle were obtained from a right parasternal short-axis view by positioning the M-mode cursor perpendicular to the interventricular septum and the left ventricular posterior wall, bisecting the left ventricle into approximately symmetric halves. The following M-mode measurements were recorded: left ventricular internal diameter at end-systole (LVIDs; cm) and end-diastole (LVIDd; cm); interventricular septal thickness at end-systole (IVSs; cm) and end-diastole (IVSd; cm); and left ventricular posterior wall thickness at end-systole (LVPWs; cm) and end-diastole (LVPWd; cm). Simultaneous electrocardiographic tracing was used only for cardiac-cycle timing during M-mode measurements, particularly to identify end-diastole at the onset of the QRS complex and end-systole at the point of maximal anterior motion of the interventricular septum. Continuous ECG monitoring and formal arrhythmia assessment were not performed during the serial echocardiographic examinations.

##### Functional indices

The following functional indices were calculated from the M-mode echocardiographic measurements. Fractional shortening (FS%) was calculated according to previously described methods [21, 26] using the following formula: FS% = [(LVIDd − LVIDs) / LVIDd] × 100, where LVIDd and LVIDs are the left ventricular internal diameters at end-diastole and end-systole, respectively. Left ventricular end-diastolic volume (LVEDV) and end-systolic volume (LVESV) were derived from linear M-mode measurements using the Teichholz formula [27] as follows: LVEDV = [7.0 × (LVIDd)³] / [2.4 + LVIDd] and LVESV = [7.0 × (LVIDs)³] / [2.4 + LVIDs]. Stroke volume (SV; mL) was calculated as SV = LVEDV − LVESV, and ejection fraction (EF%) was calculated as EF% = [(LVEDV − LVESV) / LVEDV] × 100.

#### Statistical analysis

Statistical analyses were performed using a commercial software package (SPSS for Windows, version 16.0; SPSS Inc., Chicago, IL, USA). Data were tested for normality using the Shapiro–Wilk test. As the data followed a normal distribution, results are presented as mean ± standard deviation (SD) for each variable at each time point.

A general linear model (GLM) with repeated measures was used to assess the effects of treatment (between-subject factor), time (within-subject factor), and their interaction on echocardiographic parameters. Post-hoc comparisons were performed to evaluate differences both within each treatment over time and between treatment groups at each time point. Wilks’ lambda multivariate test was used to evaluate within-subject (time) effects and time × treatment interactions. When a significant main effect or interaction was detected, univariate repeated-measures ANOVA with sphericity assumed and Bonferroni-adjusted pairwise comparisons were applied to identify differences between time points and treatment groups. For all statistical analyses, *P* < 0.05 was considered statistically significant. Effect sizes for between-group differences at each time point were calculated as eta squared (η²), using the ratio of between-group sum of squares to total sum of squares.

## Results

Repeated-measures GLM analysis revealed significant treatment × time effects for several M-mode indices, including LVIDs/LVIDd, IVSs/IVSd, LVPWs/LVPWd, LVEDV, LVESV, SV, EF%, and FS% (*P* < 0.05). The largest between-group effect sizes were observed from T15 to T60, particularly for LVESV (η² = 0.652–0.863), SV (η² = 0.635–0.774), EF% (η² = 0.501–0.741), and FS% (η² = 0.526–0.726). Post-hoc comparisons showed that these differences were not uniform across treatments and time points. No significant changes over time were detected in the control group (CG) for any of the echocardiographic variables studied.

Medetomidine (MG) produced transient echocardiographic changes, with an increase in LVIDs and reductions in selected conventional systolic indices, particularly EF% and FS%, mainly between 15 and 60 min after administration, compared with baseline and/or the control group. Butorphanol (BG) showed variable, parameter-specific effects, with some significant differences detected at certain time points compared with the CG. The combination (MBG) showed distinct time-dependent changes, with several M-mode variables differing significantly from those of the other treatment groups, predominantly between 15 and 60 min.

### Left ventricular internal diameter end-systole (LVIDs, cm)

**Fig. 1:**
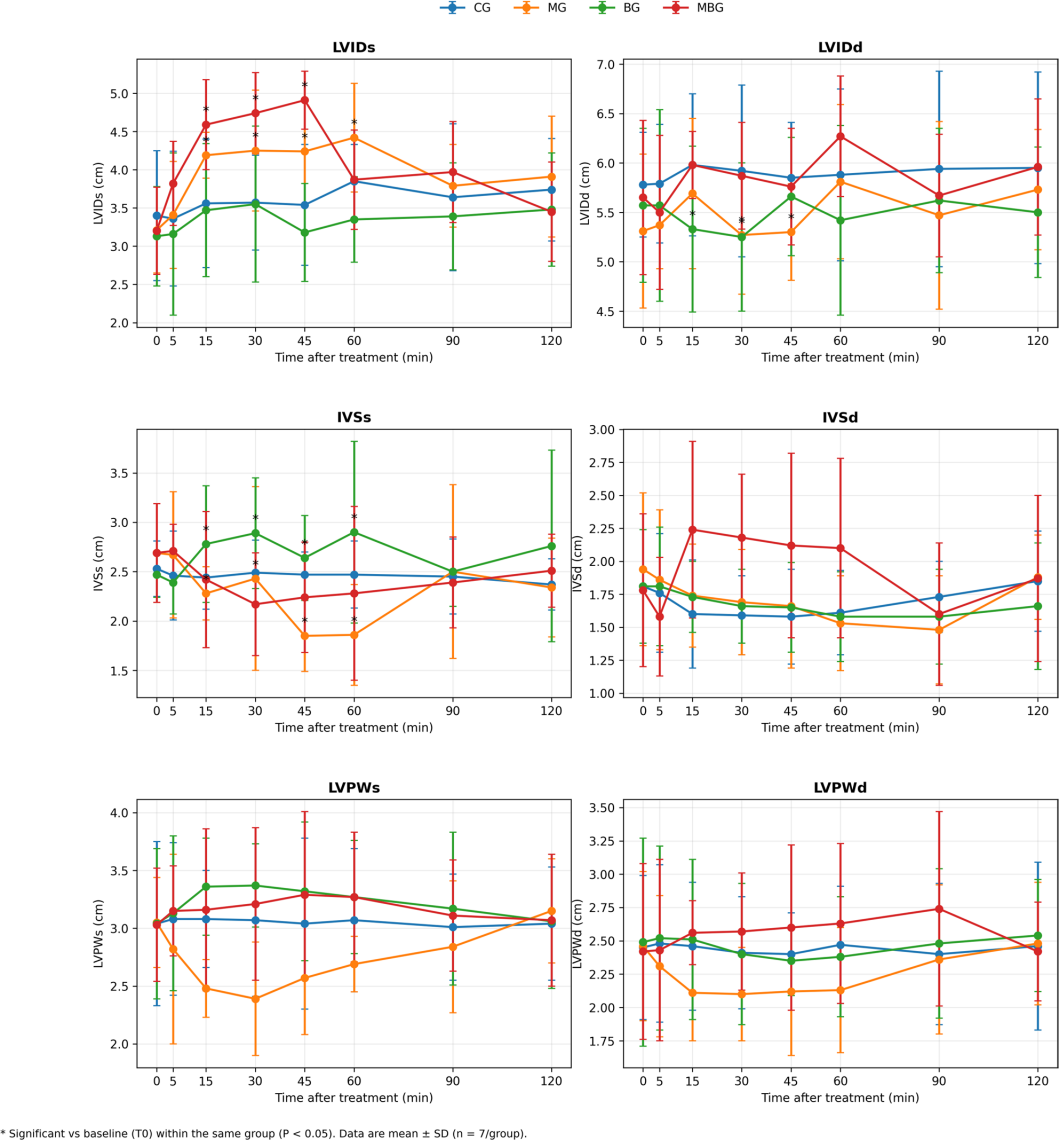
Changes in left ventricular M-mode echocardiographic dimensions in clinically healthy standing donkeys following intravenous administration of different sedative protocols. Animals were allocated to a control group (CG, saline), a medetomidine group (MG, 10 µg/kg), a butorphanol group (BG, 50 µg/kg), and a medetomidine–butorphanol group (MBG, 10 µg/kg + 50 µg/kg). Measurements were obtained from a right parasternal short-axis view at baseline (T0) and at 5, 15, 30, 45, 60, 90, and 120 min after treatment. In the MBG, T5 was obtained before butorphanol administration and was not interpreted as representing the combined effect. **A** Left ventricular internal diameter at end-systole (LVIDs); **(B)** Left ventricular internal diameter at end-diastole (LVIDd); **(C)** Interventricular septal thickness at end-systole (IVSs); **(D)** Interventricular septal thickness at end-diastole (IVSd); **(E)** Left ventricular posterior wall thickness at end-systole (LVPWs); **(F)** Left ventricular posterior wall thickness at end-diastole (LVPWd). Data are presented as mean ± SD (*n* = 15 per group). Asterisks indicate significant differences versus baseline (T0) within the same group (*P* < 0.05) and are placed on the corresponding group data points (no asterisks for CG)

LVIDs increased significantly in the MG at T15, T30, T45, and T60 compared with baseline (T0) within the same group (*P* = 0.036, 0.025, 0.024, and 0.045, respectively; Fig. 1A**).** At these time points, LVIDs in the MG were also significantly higher than those in the CG (*P* = 0.002, 0.001, 0.001, and 0.046, respectively). In the BG, LVIDs did not differ significantly from the CG at any time point. In contrast, in the MBG, LVIDs significantly increased at T15, T30, and T45 compared with T0 within the same group (*P* = 0.041, 0.036, and 0.020, respectively), and values were also significantly higher than those in the CG at the corresponding time points (*P* = 0.031, 0.022, and 0.015, respectively; Table 1**).**

**Table 1:** Echocardiographic measurements in donkeys at different time points (mean ± SD; *n* = 15 per group)

Variable	Group	T0	T5	T15	T30	T45	T60	T90	T120
LVIDs (cm)	**CG**	3.40 ± 0.85	3.36 ± 0.88	3.56 ± 0.84 ^a^	3.57 ± 0.62 ^a^	3.54 ± 0.79 ^a^	3.85 ± 0.48 ^a^	3.64 ± 0.96	3.74 ± 0.67
	**MG**	3.21 ± 0.56	3.41 ± 0.70	**4.19 ± 0.30** ^b, *^	**4.25 ± 0.79** ^b, *^	**4.24 ± 0.69** ^b, *^	**4.42 ± 0.71** ^b, *^	3.79 ± 0.54	3.91 ± 0.79
	**BG**	3.13 ± 0.65	3.16 ± 1.06	3.47 ± 0.87 ^a^	3.55 ± 1.02 ^a^	3.18 ± 0.64 ^a^	3.35 ± 0.56 ^a^	3.39 ± 0.70	3.48 ± 0.74
	**MBG**	3.20 ± 0.57	3.82 ± 0.55	**4.59 ± 0.59** ^b, *^	**4.74 ± 0.53** ^b, *^	**4.91 ± 0.38** ^b, *^	3.87 ± 0.65 ^a^	3.97 ± 0.66	3.45 ± 0.65
LVIDd (cm)	**CG**	5.78 ± 0.53	5.79 ± 0.60	5.98 ± 0.72 ^a^	5.92 ± 0.87 ^a^	5.85 ± 0.56 ^a^	5.88 ± 0.87 ^a^	5.94 ± 0.99	5.95 ± 0.97
	**MG**	5.31 ± 0.78	5.37 ± 0.44	5.69 ± 0.76 ^a^	**5.27 ± 0.60** ^b, *^	**5.30 ± 0.49** ^b, *^	5.81 ± 0.78 ^a^	5.47 ± 0.95	5.73 ± 0.61
	**BG**	5.57 ± 0.78	5.57 ± 0.97	**5.33 ± 0.84** ^b, *^	**5.25 ± 0.75** ^b, *^	5.66 ± 0.60 ^a^	5.42 ± 0.96 ^a^	5.62 ± 0.73	5.50 ± 0.66
	**MBG**	5.65 ± 0.78	5.50 ± 0.78	5.98 ± 0.34 ^a^	5.87 ± 0.54 ^a^	5.76 ± 0.59 ^a^	**6.27 ± 0.61** ^**b**^	5.67 ± 0.62	5.96 ± 0.69
IVSs (cm)	**CG**	2.53 ± 0.28	2.46 ± 0.45	2.44 ± 0.32 ^a^	2.49 ± 0.33 ^a^	2.47 ± 0.23 ^a^	2.47 ± 0.34 ^a^	2.45 ± 0.38	2.37 ± 0.26
	**MG**	2.69 ± 0.50	2.67 ± 0.64	**2.28 ± 0.27** ^a, *^	**2.43 ± 0.93** ^a, *^	**1.85 ± 0.36** ^a, *^	**1.86 ± 0.51** ^a, *^	2.50 ± 0.88	2.34 ± 0.50
	**BG**	2.47 ± 0.23	2.39 ± 0.32	**2.78 ± 0.59** ^b, *^	**2.89 ± 0.56** ^b, *^	**2.64 ± 0.43** ^b, *^	**2.90 ± 0.92** ^b, *^	2.50 ± 0.35	2.76 ± 0.97
	**MBG**	2.69 ± 0.50	2.71 ± 0.27	2.42 ± 0.69 ^a^	**2.17 ± 0.52** ^**b**^	2.24 ± 0.56 ^a^	2.28 ± 0.88 ^a^	2.39 ± 0.46	2.51 ± 0.37
IVSd (cm)	**CG**	1.81 ± 0.43	1.76 ± 0.45	1.60 ± 0.41 ^a^	1.59 ± 0.30 ^a^	1.58 ± 0.36 ^a^	1.61 ± 0.32 ^a^	1.73 ± 0.27	1.85 ± 0.38
	**MG**	1.94 ± 0.58	1.86 ± 0.53	1.74 ± 0.39 ^a^	1.69 ± 0.40 ^a^	1.66 ± 0.47 ^a^	1.53 ± 0.36 ^a^	1.48 ± 0.41	1.88 ± 0.32
	**BG**	1.81 ± 0.43	1.81 ± 0.45	1.73 ± 0.27 ^a^	1.66 ± 0.28 ^a^	1.65 ± 0.34 ^a^	1.58 ± 0.34 ^a^	1.58 ± 0.36	1.66 ± 0.48
	**MBG**	1.78 ± 0.58	1.58 ± 0.45	**2.24 ± 0.67** ^**b**^	**2.18 ± 0.48** ^**b**^	**2.12 ± 0.70** ^**b**^	**2.10 ± 0.68** ^**b**^	1.60 ± 0.54	1.87 ± 0.63
LVPWs (cm)	**CG**	3.04 ± 0.71	3.08 ± 0.66	3.08 ± 0.42 ^a^	3.07 ± 0.66 ^a^	3.04 ± 0.74 ^a^	3.07 ± 0.62 ^a^	3.01 ± 0.46	3.04 ± 0.49
	**MG**	3.05 ± 0.39	2.82 ± 0.82	2.48 ± 0.25 ^a, b^	2.39 ± 0.49 ^a, b^	2.57 ± 0.49 ^a^	2.69 ± 0.24 ^a^	2.84 ± 0.57	3.15 ± 0.45
	**BG**	3.04 ± 0.65	3.13 ± 0.67	**3.36 ± 0.42** ^**b**^	**3.37 ± 0.36** ^**b**^	**3.32 ± 0.60** ^**b**^	**3.27 ± 0.49** ^**b**^	3.17 ± 0.66	3.06 ± 0.58
	**MBG**	3.03 ± 0.49	3.15 ± 0.39	3**.16 ± 0.70** ^**b**^	3.21 ± 0.66 ^a^	3.29 ± 0.72 ^a^	3.27 ± 0.56 ^a^	3.11 ± 0.48	3.07 ± 0.57
LVPWd (cm)	**CG**	2.45 ± 0.54	2.48 ± 0.59	2.46 ± 0.48 ^a^	2.41 ± 0.42 ^a^	2.40 ± 0.31 ^a^	2.47 ± 0.44 ^a^	2.40 ± 0.53	2.46 ± 0.63
	**MG**	2.46 ± 0.56	2.31 ± 0.53	2.11 ± 0.36 ^a^	2.10 ± 0.35 ^a^	2.12 ± 0.48 ^a^	2.13 ± 0.47 ^a^	2.36 ± 0.56	2.48 ± 0.46
	**BG**	2.49 ± 0.78	2.52 ± 0.69	2.51 ± 0.60 ^a^	2.40 ± 0.53 ^a^	2.35 ± 0.26 ^a^	2.38 ± 0.45 ^a^	2.48 ± 0.56	2.54 ± 0.42
	**MBG**	2.42 ± 0.66	2.43 ± 0.68	**2.56 ± 0.24** ^**b**^	**2.57 ± 0.44** ^**b**^	**2.60 ± 0.62** ^**b**^	**2.63 ± 0.60** ^**b**^	2.74 ± 0.73	2.42 ± 0.37

Data are presented as mean ± standard deviation (SD)

*CG *control group, *MG *medetomidine group, *BG *butorphanol group, *MBG *medetomidine–butorphanol group, *LVESV *left ventricular end-systolic volume, *LVEDV *left ventricular end-diastolic volume, *SV *stroke volume, *EF *ejection fraction, *FS *fractional shortening

T0 represents baseline, and subsequent time points represent post-treatment measurements. Bold values marked with an asterisk indicate statistically significant differences from baseline (T0) within the same treatment group (*P < 0.05). Within each time point (column), values sharing at least one superscript letter (a, b, c) are not significantly different, whereas values with no letters in common differ significantly (Bonferroni-adjusted *P* < 0.05)

### Left ventricular internal diameter end-diastole (LVIDd, cm)

In the MG, LVIDd was significantly lower at T30 and T45 compared with T0 (*P* = 0.023 and *P* = 0.041, respectively; Fig. 1B) and was also significantly lower than in the CG at the corresponding time points (*P* < 0.05; Table 1). In the BG, LVIDd decreased significantly at T15 and T30 compared with T0 (*P* < 0.05) and was significantly lower than that of the CG at the same time points (*P* < 0.05; Table 1). In the MBG, no significant within-group changes in LVIDd were observed; however, LVIDd was significantly higher than in the other groups at T60 (*P* = 0.03) and showed a tendency to increase relative to T0 (*P* = 0.053; Table 1**).**

### Interventricular septal thickness at end-systole (IVSs; cm)

Time had a significant effect on IVSs in the MG and BG. In the MG, IVSs was significantly reduced at T15, T30, T45, and T60 compared with baseline (T0) (*P* < 0.01; Fig. 1C**).** In contrast, IVSs values in the BG were significantly increased at T15, T30, T45, and T60 compared with T0. No significant within-group changes were detected in the CG or MBG. Between groups, BG showed significantly higher IVSs than the CG, MG, and MBG groups at T15, T45, and T60. At T30, significant between-group differences were detected; BG and MBG differed significantly from CG and MG, while BG and MBG did not differ significantly from each other **(**Table 1**).** The time × treatment interaction was significant (Wilks’ lambda, *P* < 0.001), mainly from T15 to T60.

### Interventricular Septal Thickness at end-diastole (IVSd, cm)

Significant treatment differences in IVSd were observed at T15, T30, T45, and T60 (*P* < 0.05; Fig. 1D). During this period, IVSd values in the MG and BG remained comparable to the CG. In contrast, MBG exhibited significantly higher IVSd values than the CG, MG, and BG groups at T15, T30, T45, and T60 **(**Table 1**).** The time × treatment interaction was significant (*P* < 0.001), mainly from T15 to T60.

### Left ventricular posterior wall thickness at end-systole (LVPWs, cm)

At T15, T30, T45, and T60, significant treatment differences were observed in LVPWs (*P* = 0.013; Fig. 1E). At T15, LVPWs’ values were significantly higher in the BG and MBG than in the CG (*P* = 0.026 and *P* = 0.03, respectively), whereas the MG did not differ significantly from the other groups. At T30, BG showed significantly higher LVPWs than CG and MBG. At T45 and T60, BG had significantly higher LVPWs than the other groups (Table 1**).**

### Left ventricular posterior wall thickness at end-diastole (LVPWd, cm)

Significant treatment differences in LVPWd were observed from T15 to T60 (*P* < 0.05; Fig. 1F). At T15, T30, T45, and T60, LVPWd was significantly higher in the MBG than in the CG, MG, and BG groups (*P* = 0.040, 0.038, 0.035, and 0.030, respectively). In contrast, MG showed numerically lower LVPWd values during the same period, but it did not differ significantly from the CG or BG (Table 1**).**

### Left ventricular volume at end-systole (LVESV, mL)

**Fig. 2:**
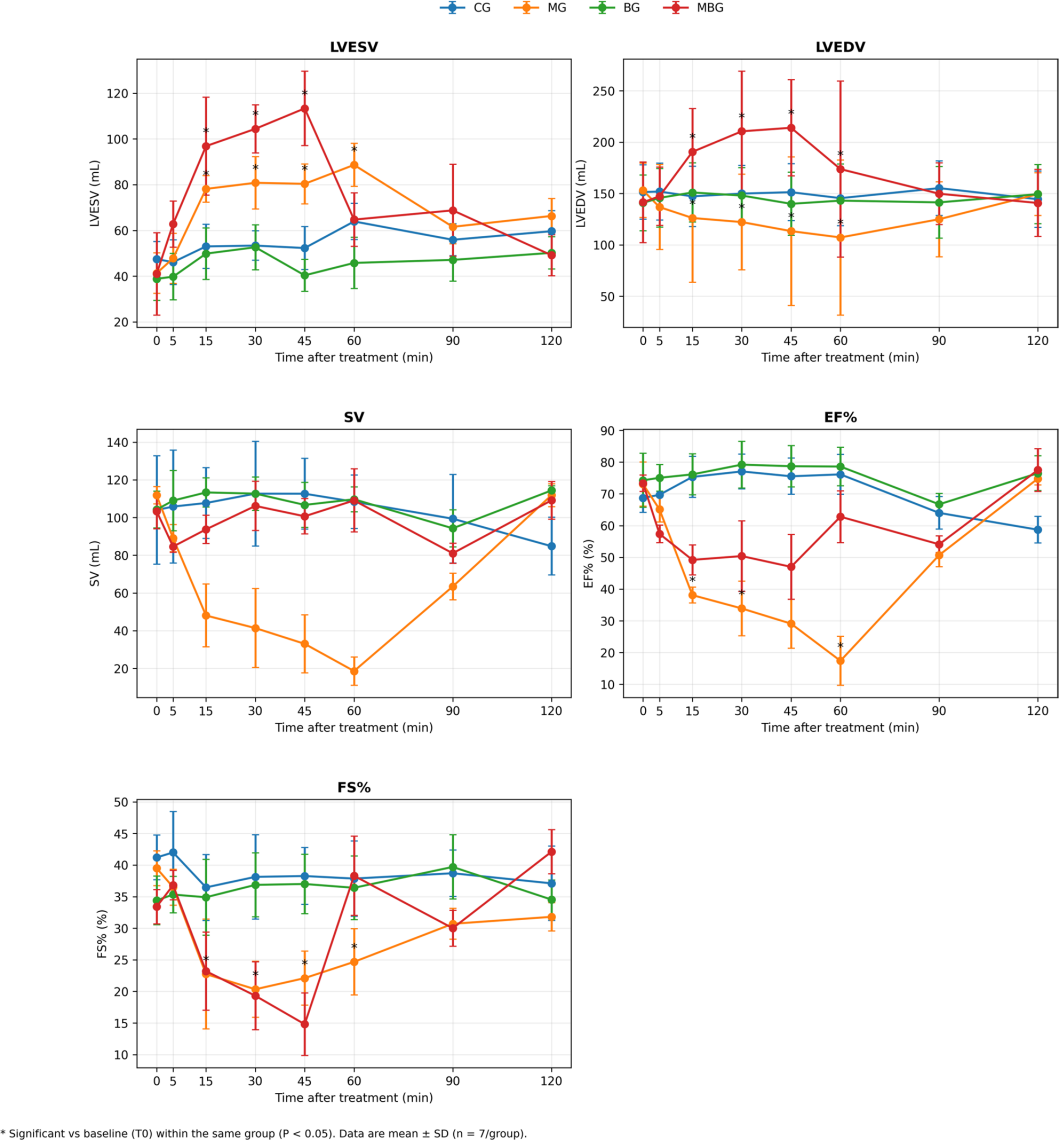
Changes in left ventricular systolic indices obtained by echocardiography in clinically healthy standing donkeys after intravenous administration of different sedative protocols. Animals were allocated to a control group (CG, saline), a medetomidine group (MG, 10 µg/kg), a butorphanol group (BG, 50 µg/kg), and a medetomidine–butorphanol group (MBG, 10 µg/kg + 50 µg/kg). Measurements were recorded at baseline (T0) and at 5, 15, 30, 45, 60, 90, and 120 min after drug administration. In the MBG, T5 was obtained before butorphanol administration and was not interpreted as representing the combined effect. **A** left ventricular end-systolic volume (LVESV); **(B)** left ventricular end-diastolic volume (LVEDV); **(C)** stroke volume (SV); **(D)** ejection fraction (EF%); (**E)** fractional shortening (FS%). Data are presented as mean ± SD (*n* = 15 per group). Asterisks indicate significant differences versus baseline (T0) within the same group (*P* < 0.05) and are placed on the corresponding group data points (no asterisks for CG)

LVESV increased significantly in the MG from T15 to T60 compared with baseline. In the MBG, LVESV increased significantly from T15 to T45 compared with baseline, followed by a return toward baseline at T60. In the BG, LVESV did not differ significantly from baseline at any post-treatment time point (Fig. 2A; Table 2**).**

**Table 2:** Calculated echocardiographic indices in donkeys at different time points (mean ± SD; *n* = 15 per group)

Variable	Group	T0	T5	T15	T30	T45	T60	T90	T120
LVESV (mL)	**CG**	47.44 ± 7.64	46.10 ± 9.74	52.99 ± 9.62 ^a^	53.35 ± 6.41 ^a^	52.28 ± 9.42 ^a^	63.91 ± 7.9 ^a^	55.89 ± 7.00	59.64 ± 8.94
	**MG**	41.27 ± 8.86	47.77 ± 10.96	**78.14 ± 5.80** ^b, *^	**80.81 ± 11.46** ^b, *^	**80.36 ± 8.72** ^b, *^	**88.63 ± 9.4** ^b, *^	61.56 ± 7.41	66.31 ± 7.66
	**BG**	38.82 ± 9.40	39.73 ± 10.13	**49.83 ± 11.27** ^**a**^	**52.63 ± 9.83** ^**a**^	**40.34 ± 6.95** ^**a**^	45.77 ± 11.2 ^a^	47.10 ± 9.28	50.17 ± 7.09
	**MBG**	40.96 ± 17.97	62.73 ± 10.09	**96.84 ± 21.42** ^c, *^	**104.41 ± 10.50** ^c, *^	**113.35 ± 16.3** ^c, *^	64.71 ± 11.7 ^a^	68.76 ± 20.1	49.14 ± 8.94
LVEDV (mL)	**CG**	151.44 ± 26.43	151.90 ± 27.66	147.26 ± 29.33 ^a^	149.95 ± 27.48 ^a^	151.3 ± 27.6 ^a^	145.5 ± 26.9 ^a^	155.3 ± 26.6	144.4 ± 27.4
	**MG**	153.07 ± 26.43	136.81 ± 41.40	**126.20 ± 62.50** ^a, *^	**122.21 ± 46.60** ^a, *^	**113.4 ± 72.3** ^a, *^	**107.24 ± 75.49** ^a, *^	124.9 ± 36.5	149.4 ± 20.9
	**BG**	140.88 ± 27.16	145.76 ± 28.51	151.03 ± 28.90 ^a^	148.04 ± 27.12 ^a^	140 ± 30.7 ^a^	143.08 ± 35.6 ^a^	141.3 ± 34.9	149.4 ± 28.8
	**MBG**	141.44 ± 39.32	147.33 ± 28.29	**190.55 ± 42.29** ^b, *^	**210.56 ± 58.58** ^b, *^	**214 ± 46.8** ^b, *^	**173.77 ± 85.8** ^b, *^	149 ± 29.95	140.8 ± 32.7
SV (mL)	**CG**	104.00 ± 28.75	105.80 ± 29.89	94.27 ± 18.72 ^a^	96.60 ± 27.74 ^a^	99.02 ± 18.8 ^a^	81.59 ± 14.2 ^a^	99.37 ± 23.5	84.80 ± 15.3
	**MG**	111.80 ± 4.52	89.04 ± 7.16^*^	**48.06 ± 16.66** ^b, *^	**41.40 ± 20.97** ^b, *^	**33.04 ± 15.32** ^b, *^	**18.61 ± 7.5** ^b, *^	63.34 ± 7.1^*^	83.09 ± 6.1^*^
	**BG**	102.09 ± 9.71	106.03 ± 15.96	101.20 ± 7.66 ^a^	95.41 ± 8.84 ^a^	99.66 ± 11.93 ^a^	97.31 ± 6.6 ^a^	94.20 ± 9.84	99.2 ± 2.47
	**MBG**	100.48 ± 9.29	84.60 ± 2.96	93.71 ± 7.49 ^a, b^	106.15 ± 13.01 ^a, b^	**100.65 ± 9.37** ^**a, b**^	**109.06 ± 16.7** ^**a, b**^	80.24 ± 5.29	91.66 ± 9.94
EF (%)	**CG**	68.7 ± 4.53	69.7 ± 4.95	64.0 ± 6.46 ^a^	64.4 ± 5.47 ^a^	65.5 ± 5.7 ^a^	**56.1 ± 6.28** ^**b**^	64.0 ± 5.12	58.7 ± 4.20
	**MG**	73.06 ± 6.96	65.1 ± 3.94	**38.1 ± 2.49** ^b, *^	**33.9 ± 8.58** ^b, *^	**29.1 ± 7.7** ^b, *^	**17.4 ± 7.68** ^a, *^	50.7 ± 3.68	55.6 ± 2.02
	**BG**	72.4 ± 8.54	72.7 ± 4.18	67.0 ± 6.45 ^a^	64.5 ± 7.34 ^a^	71.2 ± 6.49 ^a^	68.0 ± 6.02 ^b^	66.7 ± 3.44	66.4 ± 5.50
	**MBG**	71.0 ± 2.55	57.4 ± 2.74	**49.2 ± 4.71** ^c, *^	**50.4 ± 11.08** ^c, *^	**47.0 ± 10.2** ^c, *^	**62.8 ± 8.14** ^**c**^	54.1 ± 2.64	65.1 ± 6.72
FS (%)	**CG**	41.2 ± 3.55	42.0 ± 6.47	36.46 ± 5.22 ^a^	38.13 ± 6.68 ^a^	38.26 ± 4.5 ^a^	37.86 ± 5.97 ^a^	38.7 ± 3.68	37.1 ± 5.89
	**MG**	39.5 ± 2.75	36.5 ± 2.85	**22.73 ± 8.69** ^b, *^	**20.33 ± 4.45** ^b, *^	**22.1 ± 4.3** ^b, *^	**24.67 ± 5.23** ^b, *^	30.7 ± 2.44	31.8 ± 2.25
	**BG**	34.40 ± 3.86	35.33 ± 2.89	34.9 ± 5.99 ^a^	36.86 ± 5.05 ^a^	37 ± 4.70 ^a^	36.40 ± 5.03 ^a^	39.7 ± 5.08	34.53 ± 3.11
	**MBG**	33.40 ± 2.72	36.80 ± 2.33	**23.2 ± 6.19** ^b, *^	**19.3 ± 5.39** ^b, *^	**14.8 ± 4.94** ^c, *^	38.3 ± 6.28 ^a^	30.0 ± 2.85	42.1 ± 3.51

Data are presented as mean ± standard deviation (SD)

*CG *control group, *MG *medetomidine group, *BG *butorphanol group, *MBG *medetomidine–butorphanol group, *LVESV *left ventricular end-systolic volume, *LVEDV *left ventricular end-diastolic volume, *SV *stroke volume, *EF *ejection fraction, *FS *fractional shortening

T0 represents baseline, and subsequent time points represent post-treatment measurements. Bold values marked with an asterisk indicate statistically significant differences from baseline (T0) within the same treatment group (*P < 0.05). Within each time point (column), values sharing at least one superscript letter (a, b, c) are not significantly different, whereas values with no letters in common differ significantly (Bonferroni-adjusted *P* < 0.05)

### Left ventricular volume at end-diastole (LVEDV mL)

LVEDV was significantly affected by time in the MG and MBG (*P* = 0.026 and 0.002, respectively; Fig. 2B**).** In the MG, LVEDV showed a decreasing trend from T15 to T60, whereas in the MBG, LVEDV increased during the same interval. Based on the post-hoc comparisons shown in Table 2, LVEDV in the MBG was significantly higher than in the other groups from T15 to T60. The time × treatment interaction was significant from T15 to T60 (*P* < 0.05).

### Stroke volume (SV, mL)

Stroke volume SV decreased significantly in the MG at all post-treatment time points compared with baseline. Between-group post-hoc comparisons showed that SV in the MG was lower than in the CG from T15 to T60. In the BG, SV remained comparable to the CG. SV values were recalculated from LVEDV and LVESV and interpreted as secondary load-dependent variables; therefore, conclusions regarding increased SV in the MBG were avoided **(**Fig. 2C; Table 2**).**

### Ejection fraction (EF%)

EF% was significantly affected by time in the MG and MBG (*P* = 0.001; Fig. 2D). In the MG, EF% decreased significantly from T15 to T60 compared with baseline and was lower than the corresponding values in the CG and BG, as indicated by the post-hoc annotations in Table 2. In the MBG, EF% decreased significantly from T15 to T45 compared with baseline, followed by partial recovery at T60. In contrast, EF% remained relatively stable in the CG and BG throughout the observation period. The time × treatment interaction was significant from T15 to T60 (*P* < 0.05), indicating that the EF% response differed among treatment groups over time.

### Fractional shortening (FS%)

FS% was significantly affected by time in the MG and MBG (*P* = 0.001 for both; Fig. 2E**).** In the MG, FS% decreased from T15 to T60 compared with baseline, indicating a transient reduction in conventional systolic indices. In the MBG, FS% decreased significantly from T15 to T45 compared with baseline, followed by recovery at T60. However, because FS% is derived directly from LVIDd and LVIDs and is influenced by loading conditions, these changes were interpreted cautiously and were not considered evidence of enhanced intrinsic myocardial contractility. The time × treatment interaction was significant from T15 to T60 (*P* < 0.05).

## Discussion

This study showed that the three sedation protocols had distinct effects on echocardiographic parameters in donkeys. Medetomidine alone was associated with transient depression of conventional left ventricular systolic indices, whereas butorphanol alone produced only minor cardiovascular changes. The medetomidine–butorphanol combination produced marked changes in ventricular dimensions and calculated indices; however, because LVESV, SV, EF, and FS were derived from linear M-mode measurements and are influenced by loading conditions, these changes were interpreted cautiously and not as evidence of maintained or enhanced intrinsic systolic performance.

These findings are broadly consistent with previous echocardiographic observations in donkeys. Ibrahim et al. reported that epidural administration of xylazine or dexmedetomidine altered echocardiographic dimensions and cardiac indices in clinically healthy donkeys [12]. Although the route of administration and the α2-agonists differed from those used in the present study, their findings support the concept that α2-agonists can modify cardiac dimensions and derived functional indices in donkeys.

Evidence from donkey sedation studies is also consistent with the present findings. Intramuscular detomidine combined with butorphanol has been reported to provide satisfactory standing sedation in donkeys, with only moderate, transient cardiopulmonary changes and no clinically relevant compromise in clinically healthy animals [28]. Although that study did not primarily evaluate echocardiographic indices, it supports the interpretation that α2-agonist/opioid combinations may be tolerated in healthy donkeys when appropriate monitoring is used.

The cardiovascular effects observed after medetomidine alone also agree with the known actions of α2-agonists in equids, including bradycardia, increased systemic vascular resistance, and reduced cardiac output [29–31]. These mechanisms may explain the transient reduction in ventricular volumes, SV, EF%, and FS% observed in the present study. Similar cardiovascular responses have been reported in horses and mules, although the magnitude of change depends on dose, route of administration, depth of sedation or anaesthesia, and species-specific sensitivity [30, 32]. Compared with reports describing more pronounced cardiovascular depression under general anaesthesia or after higher α2-agonist doses, the changes observed in the present standing donkeys were relatively mild and reversible, suggesting that both protocol and depth of sedation strongly influence echocardiographic interpretation.

The increased ventricular dimensions observed after the MBG are best explained by altered loading conditions rather than by intrinsic myocardial hypercontractility. α2-agonist-induced bradycardia may prolong diastolic filling time, thereby increasing preload and ventricular filling [29–31, 33].

The change in SV within the MBG was less pronounced than the changes observed in ventricular dimensions and calculated volumes. Therefore, the response in this group is better interpreted as a load-dependent change rather than a marked increase in SV or enhanced myocardial performance [27].

Because EF% and FS% are load-dependent indices, they may appear preserved or increased when preload rises, or effective afterload is reduced. A similar dissociation between ventricular enlargement and apparently enhanced systolic indices has been reported in volume-overload states, such as chronic mitral regurgitation in dogs and horses [16, 34]. Therefore, the relatively higher EF and FS observed in the MBG compared with the MG should not be interpreted as a true positive inotropic effect.

The increase in LVESV in the MBG should also be interpreted cautiously. LV volumes in this study were calculated from linear left ventricular dimensions; therefore, moderate changes in systolic diameter may be amplified when expressed as calculated volume [27]. This is important because an increase in LVESV could otherwise be misinterpreted as impaired systolic emptying. Therefore, changes in LVESV, SV, EF%, and FS% should be interpreted cautiously and should not be used alone to infer intrinsic myocardial contractility.

By comparison, butorphanol alone produced the most cardiovascularly stable profile. This agrees with reports in horses [35] and dogs [36–38] showing that butorphanol has limited direct cardiodepressant effects at clinically relevant doses, usually causing only modest changes in heart rate and arterial blood pressure. Together with the available donkey data, these findings support the use of butorphanol as a comparatively cardiovascular-sparing sedative option in clinically healthy donkeys [28]. However, this interpretation should be limited to clinically healthy animals, as responses may differ in donkeys with myocardial dysfunction, valvular disease, dehydration, pain, or systemic illness.

Findings in other domestic species provide useful mechanistic support, although direct extrapolation to donkeys should be cautious. In dogs, butorphanol–medetomidine or butorphanol–dexmedetomidine protocols have been associated with changes in cardiac output and ventricular dimensions, with the magnitude of these effects influenced by α2-agonist dose [33, 36, 39]. In equids, α2-agonists are known to induce bradycardia, increased vascular resistance, and reduced cardiac output, while the severity of echocardiographic changes varies with dose, route, and anaesthetic depth [32–35]. The milder changes observed in the present standing donkeys probably reflect differences in protocol, species, and depth of sedation.

Importantly, most echocardiographic values remained within published reference limits for donkeys despite statistically significant differences between protocols or time points [20, 21, 40, 41]. This distinction is clinically relevant because statistical significance does not necessarily indicate pathological cardiac dysfunction. The observed changes are therefore best regarded as transient functional adaptations to sedation-related alterations in loading conditions rather than evidence of structural cardiac disease. Nevertheless, animals with subclinical disease may have less cardiovascular reserve, and similar changes could become clinically relevant in compromised patients.

Clinically, butorphanol alone appeared to be the most cardiovascularly sparing protocol among those tested. Medetomidine alone should be used cautiously in older donkeys or animals with suspected myocardial or valvular disease because of its transient depressive effect on systolic performance and stroke volume. The MBG may be useful in clinically healthy donkeys when enhanced standing sedation is required; however, the observed echocardiographic changes should be interpreted as load-dependent rather than evidence of preserved or enhanced intrinsic myocardial performance. Cardiovascular monitoring remains advisable, particularly in animals with suspected cardiac compromise.

Several limitations of the present study should be acknowledged. The number of animals in each treatment group was relatively small, which may limit the statistical power to detect subtle differences and restrict the generalizability of the findings. Although an electrocardiographic tracing was used for cardiac-cycle timing during M-mode measurements, continuous electrocardiogram (ECG) monitoring and formal arrhythmia analysis were not performed; therefore, potential medetomidine-induced arrhythmias could not be systematically evaluated. Echocardiographic measurements were obtained from standard right-parasternal M-mode recordings and therefore provide only one-dimensional information about left ventricular geometry; regional wall motion abnormalities and three-dimensional changes could not be assessed. Formal intra-observer reproducibility testing was not performed, which may have limited the assessment of measurement repeatability. However, all measurements were obtained by a single examiner using a standardized protocol and were averaged over three non-consecutive cardiac cycles to reduce beat-to-beat variability. Only a single dose of each protocol was evaluated; therefore, further studies are needed to assess different dose regimens, repeated administrations, and responses in donkeys with underlying cardiac disease.

In addition, invasive hemodynamic measurements (e.g., arterial blood pressure and cardiac output) were not performed, which limits the ability to distinguish primary changes in contractility from secondary changes in loading conditions. Sedation scores were not assessed in the present study, as the primary objective was to evaluate echocardiographic changes rather than sedation quality. Therefore, the relationship between sedation depth and echocardiographic alterations could not be determined. Future studies including both healthy and cardiopathic donkeys, and combining advanced echocardiographic techniques with invasive hemodynamic monitoring and sedation scoring, would be valuable to refine evidence-based recommendations for the safe use of medetomidine, butorphanol, and their combinations.

## Conclusion

Intravenous medetomidine, butorphanol, and their combination produced distinct cardiovascular effects in clinically healthy standing donkeys. Medetomidine induced transient depression of conventional systolic indices, whereas butorphanol had minimal cardiovascular impact. The MBG combination may be considered for standing sedation in clinically healthy donkeys; however, its echocardiographic effects should be interpreted cautiously as load-dependent changes, and cardiovascular monitoring remains advisable.

## Data Availability

All data supporting the findings of this study are available within the paper.
